# Meiotic Recombination Initiation in and around Retrotransposable Elements in *Saccharomyces cerevisiae*

**DOI:** 10.1371/journal.pgen.1003732

**Published:** 2013-08-29

**Authors:** Mariko Sasaki, Sam E. Tischfield, Megan van Overbeek, Scott Keeney

**Affiliations:** 1 Molecular Biology Program, Memorial Sloan-Kettering Cancer Center, New York, New York, United States of America; 2 Weill Graduate School of Medical Sciences, Cornell University, New York, New York, United States of America; 3 Tri-Institutional Training Program in Computational Biology and Medicine, Weill Cornell Medical College, New York, New York, United States of America; 4 Howard Hughes Medical Institute, Memorial Sloan-Kettering Cancer Center, New York, New York, United States of America; National Cancer Institute, United States of America

## Abstract

Meiotic recombination is initiated by large numbers of developmentally programmed DNA double-strand breaks (DSBs), ranging from dozens to hundreds per cell depending on the organism. DSBs formed in single-copy sequences provoke recombination between allelic positions on homologous chromosomes, but DSBs can also form in and near repetitive elements such as retrotransposons. When they do, they create a risk for deleterious genome rearrangements in the germ line via recombination between non-allelic repeats. A prior study in budding yeast demonstrated that insertion of a Ty retrotransposon into a DSB hotspot can suppress meiotic break formation, but properties of Ty elements in their most common physiological contexts have not been addressed. Here we compile a comprehensive, high resolution map of all Ty elements in the rapidly and efficiently sporulating *S. cerevisiae* strain SK1 and examine DSB formation in and near these endogenous retrotransposable elements. SK1 has 30 Tys, all but one distinct from the 50 Tys in S288C, the source strain for the yeast reference genome. From whole-genome DSB maps and direct molecular assays, we find that DSB levels and chromatin structure within and near Tys vary widely between different elements and that local DSB suppression is not a universal feature of Ty presence. Surprisingly, deletion of two Ty elements weakened adjacent DSB hotspots, revealing that at least some Ty insertions promote rather than suppress nearby DSB formation. Given high strain-to-strain variability in Ty location and the high aggregate burden of Ty-proximal DSBs, we propose that meiotic recombination is an important component of host-Ty interactions and that Tys play critical roles in genome instability and evolution in both inbred and outcrossed sexual cycles.

## Author Summary

Meiosis is the cell division that generates gametes for sexual reproduction. During meiosis, homologous recombination occurs frequently, initiated by DNA double-strand breaks (DSBs) made by Spo11. Meiotic recombination usually occurs between sequences at allelic positions on homologous chromosomes, but a DSB within a repetitive element (e.g., a retrotransposon) can provoke recombination between non-allelic sequences instead. This can create genomic havoc in the form of gross chromosomal rearrangements, which underlie many recurrent human mutations. It has been thought that cells minimize this risk by disfavoring DSB formation in repetitive elements, partly based on studies showing that presence of a Ty element (a yeast retrotransposon) can suppress nearby DSB activity. Whether this is a general feature of Tys has not been evaluated, however. Here, we generated a comprehensive map of Tys in the rapidly sporulating SK1 strain and examined DSB formation in and around all of these endogenous Ty elements. Remarkably, most natural Ty elements do not appear to suppress DSB formation nearby, and at least some of them increase local DSBs. These findings have implications for understanding the relationship between host and transposon, and for understanding the impact of retrotransposons on genome stability and evolution during sexual reproduction.

## Introduction

Meiosis is the specialized cell division that halves the genome complement to produce gametes for sexual reproduction. During meiosis, homologous recombination is induced by programmed DNA double-strand breaks (DSBs) made by the topoisomerase-like Spo11 protein in a reaction in which Spo11 attaches covalently to 5′ strand termini of the DSB [1]. Endonuclease cleavage releases Spo11 from the DSB ends in a covalent complex with a short oligonucleotide [2]. Subsequent resection of DSB 5′ strand ends generates 3′ single-stranded tails, which are substrates for proteins that search for a homologous DNA duplex and effect the templated repair of the break [3].

DSBs in single-copy sequences usually induce recombination between allelic segments on homologous chromosomes, which promotes pairing and accurate segregation of homologs and increases genetic diversity in gametes. However, eukaryotic genomes are replete with repetitive elements that share high sequence identity. A DSB formed in a repeat can induce recombination between non-allelic DNA segments, which can in turn result in chromosome rearrangements such as duplications, deletions, inversions or translocations [4]–[6]. In humans, such non-allelic homologous recombination (NAHR, also referred to as ectopic recombination) in the germ line contributes to non-pathogenic structural variation [7] and is linked to numerous genomic disorders [5]. NAHR is thus a driving force in genome evolution and a source of genome instability. Meiotic DSBs are distributed non-randomly across genomes [8], [9], so the propensity toward NAHR depends strongly on how likely it is that Spo11 cuts in and around repetitive elements [6].

A major class of repetitive element in *S. cerevisiae* comprises the Ty elements, ∼6-kb retrotransposons related to mammalian retroviruses [10]. Each contains an internal region encoding Gag- and Pol-like proteins required for retrotransposition, flanked by ∼330-bp long terminal repeats (LTRs). The S288C strain (source of the yeast reference genome) contains 50 Ty elements in five distinct families: 31 Ty1, 13 Ty2, 2 Ty3, 3 Ty4 and 1 Ty5 [11]. S288C also contains a much larger number of solo LTRs or LTR fragments, which likely arise from homologous recombination between the LTRs of full-length Tys [11]. The predominant families, Ty1 and Ty2, exhibit high sequence identity: >90% in pairwise comparisons within families and >70% between Ty1 and Ty2 [11], [12]. Because of their sequence similarity and dispersed distribution, Ty elements are potent sources of gross chromosomal rearrangements. Numerous studies have documented Ty-mediated NAHR induced by DSBs or replication errors in vegetatively growing cells [e.g.], [ 12]–[15].

Comparatively little is known about Ty recombination provoked by Spo11-generated DSBs in meiosis. A *URA3*-marked Ty2 element inserted in the *HIS4* promoter caused a >13-fold reduction in DSBs at this site, which is normally a strong DSB hotspot [16]. An open (nucleosome-depleted) chromatin structure is an important determinant of Spo11 hotspots [17]–[19]. The *HIS4* promoter, like most yeast promoters, displays hypersensitivity to DNase I digestion of chromatin, but the inserted Ty (which is itself resistant to nuclease digestion), converted the local chromatin structure to a nuclease-resistant state [16]. Thus, a Ty can suppress DSB formation nearby, possibly via spreading of a closed chromatin structure into the surrounding region [16]. However, although this element mimicked a spontaneous Ty insertion [20], it is in an unusual position since Tys most often integrate near tRNA genes and only rarely into RNA pol II promoters [11], where most DSB hotspots occur in yeast [19]. By direct restriction mapping and Southern blotting on chromosome III in the rapidly and efficiently sporulating *S. cerevisiae* SK1 strain, two novel Tys were identified [21]. DSBs were not detected within or adjacent to these Tys, but it remained unknown whether DSBs are infrequent in or near other natural Tys. Also, Ty elements can differ widely from one another in many of their behaviors. For example, the few Tys examined to date undergo NAHR at dissimilar frequencies, ranging from ∼10^−5^ to ∼10^−2^ per meiosis [4], [22], [23], and expression of Tys and Ty-adjacent genes varies substantially between individual elements [10], [24]. Thus, it is presently unknown whether local DSB suppression can be extrapolated to be a general feature of natural Ty elements.

Deep sequencing of the Spo11 oligos that are byproducts of DSB formation provided a high resolution DSB map and suggested that DSBs are moderately suppressed within Tys on average [19]. However, average behavior does not reveal the extent of variation between different sites. Here, we examine DSB formation in and around endogenous Ty elements in SK1 and explore how the presence of natural Ty elements affects local DSB frequency.

## Results

### Comprehensive, High-Resolution Map of Ty Elements in SK1

Full-length Ty1 and Ty2 elements were previously mapped in SK1 by microarray hybridization of genomic DNA containing Ty sequences [25]. Twenty-five Ty-containing regions were identified, but spatial resolution (ranging 1–21 kb) was not high enough for us to assess nearby DSBs. The *Saccharomyces* Genome Resequencing Project (SGRP) generated an SK1 genome assembly from shotgun sequencing combined with phylogenetic comparisons [26]. The number of Ty-containing reads led to an estimate that retrotransposons are ∼2% of the SK1 genome vs. >3% for S288C, implying SK1 has ∼30 Tys. However, the SGRP assembly and its subsequent refinement [27] did not compile all Ty elements, identify Ty families, or reveal precise Ty positions, because repetitive elements pose a computational challenge in genome assembly [26], [28], [29]. Moreover, the SK1 strain sequenced by SGRP is a homothallic, prototrophic strain (*HO, LYS2, URA3, LEU2*) related to an ancestor of the strains most widely used in meiosis research [30].

**Figure 1:**
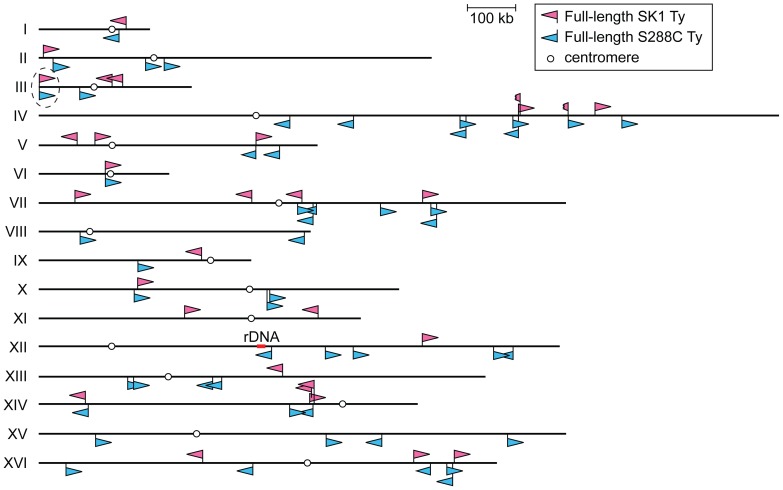
Location of Ty elements in SK1. The insertion sites and orientations of SK1 Ty elements are shown in comparison to S288C Tys (chromosomal coordinates are from S288C). Fragmented arrowheads indicate partial Ty elements. Open circles show centromeres. Dashed circle highlights the only Ty shared between the two strains.

To overcome these limitations, we took a multipronged approach to precisely map all full-length Ty elements in the Kleckner laboratory-derived SK1 lineage. We identified a total of 30 Tys and fine-mapped their positions, in most cases to single-nucleotide resolution (Figure 1 and Table 1).

**Table 1:** Location of Ty elements in SK1.

Chr	Name	Start^a^	End^a^	Family	Strand	tRNA^c^	Target seq.^d^
I	Ty*_YAR023C-UIP3_*	180826	180845	Ty1	−	+	GTTTA
II	Ty*_YBL108W-YBL107C_* f	9462^e^	9463^e^	Ty1	+	+	N.D.
III	Ty*_TEL03L-YCL073C_* f *^,^* g	1180	4322	Ty5	+	−	N.D.
III	Ty*_SRD1-MAK32_*	151729	151730	Ty2	−	+	GAATC
III	Ty*_RIM1-SYP1_*	173740	173741	Ty1	−	−	AAATC
IV	Ty*_EXG2-YDR262W-1_* h	992677	992678	Ty1	+	+	AAGAT
IV	Ty*_EXG2-YDR262W-2_* h	992677	992678	Ty1 or Ty2	−	+	N.D.
IV	Ty*_OMS1-HIM1_* i	1095501	1095502	Ty1	−	+	ATATG
IV	Ty*_FCF1-YDR341C_*	1151116^e^	1151117^e^	Ty1	+	+	GTCTA
V	Ty*_UTR2-CYC7_*	79534	79535	Ty2	−	−	AATAT
V	Ty*_URA3_*	116283	116284	Ty1^b^	+	−	CGTAC
V	Ty*_YER137C-RTR1_*	449646^e^	449647^e^	Ty1^b^	+	+	N.D.
VI	Ty*_MSH4-SPB4_*	137731	137732	Ty1	+	+	N.D.
VII	Ty*_YGL226W-VRG4_*	74930	74931	Ty2	+	+	GATAA
VII	Ty*_CGR1-SCW11_*	441007	441008	Ty1	−	+	ATAAT
VII	Ty*_ERV1-POP6_*	544849	544850	Ty1	−	+	AATAT
VII	Ty*_YGR150C-RSR1_*	794408^e^	794409^e^	Ty1	+	+	ATATT
IX	Ty*_EST3-FAA3_*	336955	336956	Ty1	−	+	GTTTT
X	Ty*_ASF1-MDV1_*	204645	204646	Ty1	+	+	AAAGG
XI	Ty*_STB6-YKL071W_*	301921	301922	Ty1	+	+	GAAGG
XI	Ty*_SIS2-YKR074W_*	578126	578127	Ty1	−	+	ATTAG
XII	Ty*_MID2-RPS25B_*	793754	793755	Ty2	+	+	ACTAT
XIII	Ty*_YMR118C-ASI1_*	504714^e^	504715^e^	Ty1	−	+	ATTAT
XIV	Ty*_CUS2-MRPL10_*	96517^e^	96521^e^	Ty1	−	+	TATAT
XIV	Ty*_NCE103-YNL035C-1_* j	560747	560748	Ty2	+	+	GAAAC
XIV	Ty*_NCE103-YNL035C-2_* j	560747	560748	Ty1 or Ty2	−	+	N.D.
XIV	Ty*_YNL035C-YNL034W_*	569954	569955	Ty3	−	+	GTTTT
XVI	Ty*_PEX25-CAR1_*	339200	339402	Ty1	−	+	–
XVI	Ty*_CLB5-THI22_*	776100	776101	Ty1	+	+	ATGAA
XVI	Ty*_KRE6-GPH1_*	859975	859976	Ty1	+	+	GTTTA

a The coordinates of Ty insertion sites are based on the S288C reference genome (the June 2008 assembly from the Saccharomyces Genome Database). When a Ty insertion site exhibits a 5-bp duplication, the third and fourth bp are used as the start and end coordinates, respectively.

b Although the family of Ty*_URA3_* and Ty*_YER137C-RTR1_* could not be determined by established criteria [11] (Figure S1D), these Tys were classified as Ty1 by Gabriel et al. (2006).

c The presence (+) or absence (−) of tRNA in the same intergenic region with a Ty is indicated.

d The target site sequence duplicated on the same strand with a Ty is indicated. N.D. indicates that the presence or absence of sequence duplication was not determined. “–” indicates that sequence duplication was not observed.

e Ty element is inserted in a novel SK1 LTR. The insertion site of the LTR is indicated.

f Ty*_YBL108W-YBL107C_* and Ty*_TEL03L-YCL073C_* are located in subtelomeric regions, which are enriched with repeated sequences and are dynamic among strains [65]. Since chromosome ends are not well defined in the SK1 genome assembly, it remains to be determined which chromosome end(s) carry these Tys.

g Ty*_TEL03L-YCL073C_* is the same as *YCLWTy5-1* in S288C.

h A full-length Ty*_EXG2-YDR262W-1_* and a fragmented Ty*_EXG2-YDR262W-2_* of >1 kb in size are located adjacent to each other.

i Ty*_OMS1-HIM1_* is a ∼1-kb fragmented Ty.

j Ty_NCE103-YNL035C-1_ is disrupted by Ty_NCE103-YNL035C-2_.

**Figure 2:**
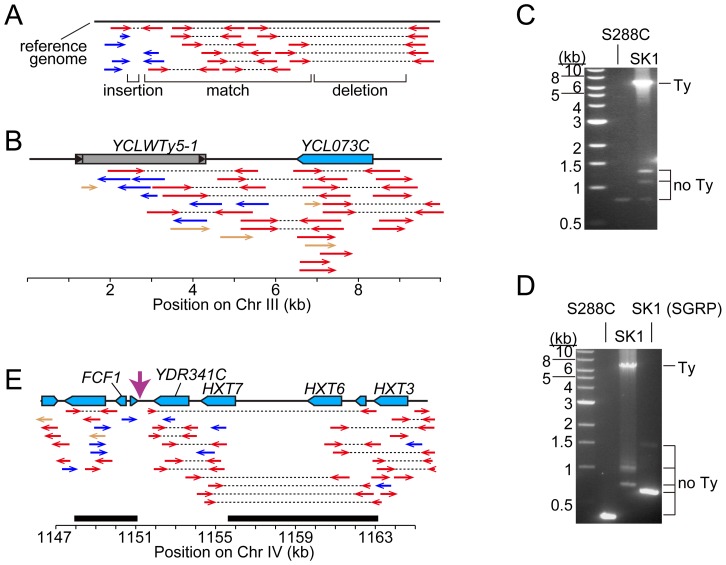
Identifying Ty insertion sites. (A) SK1-derived sequence reads aligned to the S288C genome. Red arrows connected by dotted lines represent paired ends that align near one another. Blue arrows are orphan reads whose mates are aligned inconsistently, e.g., to different chromosomes. Expected patterns are shown for a region where the SK1 genome matches the reference genome, and regions containing a deletion or insertion. (B) Snapshot of the SGRP browser in a simplified cartoon form, depicting SK1-derived reads mapped near *YCLWTy5-1*. The color scheme is as in (A), plus light brown arrows for reads whose paired ends were not sequenced. (C, D) Confirmation of Ty insertions in SK1 by PCR at *URA3* (C) or the *YMR118C*-*ASI1* intergenic region (D). Smaller bands amplified from SK1 genomic DNA are *ex vivo* deletion products from LTR-LTR recombination during PCR. (E) SK1 sequence reads mapped to the S288C genome near *FCF1*. Black bars indicate where SK1 Ty1 or Ty2 were previously mapped [25]. Vertical pink arrow shows the single Ty position mapped in this study. The tandemly duplicated gene pair of *HXT6* and *HXT7* present in S288C is a single copy in SK1, without the intervening sequence.

First, we asked whether SK1 has Tys that are present in S288C. The SGRP data consist of paired-end sequence reads with an average insert of 4–5 kb. These reads can reveal structural differences between a reference genome and the DNA source if the distance between mapped pairs is substantially larger or shorter than the average, or if orphans are present, where one read maps but its mate fails to map or maps to a different genomic region and/or multiple locations (Figure 2A). From inspection of SGRP read maps, we found only one S288C element that was also present in SK1: *YCLWTy5-1* at the left end of Chr III (Figure 1, Figure 2B and Table 1). This is the only full-length Ty5 family member in either strain, although there are Ty5 solo LTRs and LTR fragments in both (data not shown). Ty5-family insertions are found preferentially near telomeres and silent mating type loci [reviewed in 11]. *YCLWTy5-1* contains mutations rendering it nonfunctional for transposition [31], so this is an ancient Ty present in the last common ancestor of these strains. The remaining 49 S288C Tys are not present in SK1 (Figures S1A, S1B and data not shown). This manual inspection also identified eight S288C Ty sites for which SK1 has one or more Ty elements nearby, subsequently confirmed by PCR (11 Tys total; Figure S1B, Table 1 and data not shown). These novel Tys are at different positions in SK1 than in S288C and often of a different family or in opposite orientation, thus are independent integration events.

Second, we evaluated Ty1 and Ty2 sites mapped by Gabriel et al. in the Kleckner lineage [25]. Using SGRP sequence patterns plus PCR and sequencing of genomic DNA, we validated and fine-mapped 22 SK1-specific elements (Figure S1C and data not shown), but 3 sites showed no evidence of a Ty in SGRP data. Two of these reflect differences between SGRP and Kleckner SK1 strains: a spontaneous *ura3* mutation selected during derivation of the Kleckner strains and caused by Ty integration [30], [32] (Figure 2C); and a Ty1 on Chr XIII (Figure 2D and data not shown). The latter is likely a de novo, unselected integration that passed through the bottlenecks of strain derivation, demonstrating the potential for occult differences between otherwise isogenic strains. The third discrepancy is a single Ty incorrectly assigned to two separate sites on Chr IV. S288C contains a tandem duplication of similar genes encoding a hexose transporter (*HXT6* and *HXT7*) [33], but SK1 has only one *HXT* copy in this region and lacks the intervening sequence (Figure 2E and data not shown). This structural difference caused the microarray hybridization data to artifactually give two peaks from a single Ty when projected onto S288C sequence space.

**Figure 3:**
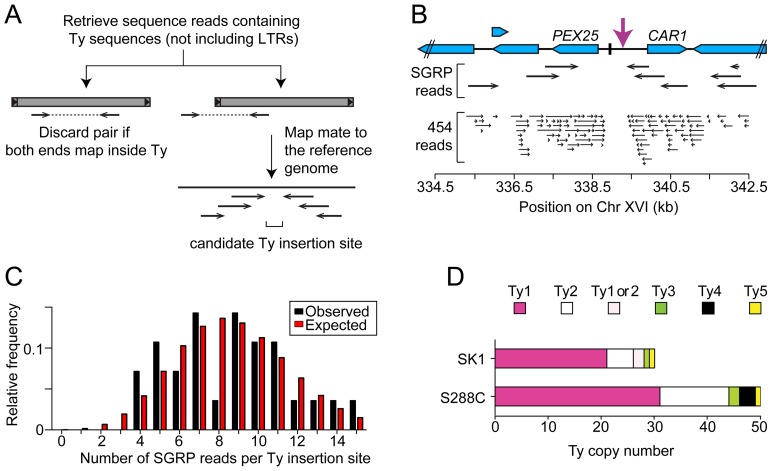
Systematic Ty mapping. (A) Mapping strategy. (B) Ty in the *PEX25*-*CAR1* intergenic region. (C) Number of SGRP reads supporting each Ty position in SK1. The observed distribution of read frequencies around each of 28 Ty sites is compared to that expected from a Poisson distribution with the same mean (λ = 8.6). (D) Copy number of Tys from different families in SK1 and S288C.

Third, we used an unbiased approach to ensure that all Ty elements were identified, using SGRP data and a paired-end genomic sequence library from NKY291, a Kleckner-lineage haploid. We retrieved sequence pairs in which one mate matched non-LTR parts of Tys, then mapped the non-Ty mate on the S288C genome (277 SGRP reads and 4,963 NYK291 reads, <1% of the total from each). Tys appear as clusters of reads pointing from both directions at the insertion site (Figures 3A and 3B). We identified all of the Tys described above, and also found an additional element on Chr II, present in both libraries and confirmed by PCR (data not shown). On average, 8.6 SGRP reads tagged each SK1-specific Ty or cluster of Tys, and the read counts matched a Poisson distribution (Figure 3C). Thus, we estimate the probability to be <0.0002 that a Ty was missed because of chance failure to recover supporting reads. The NKY291 library provided even more reads identifying each Ty (mean 158.2, range 30–304), so it is highly likely we identified all of the Tys in SK1.

### Comparison between Strains

SK1 Tys showed both conserved and non-conserved features with their S288C counterparts. Ty1 and Ty2 are the predominant families, as in S288C, and only one each of Ty3 and Ty5 are present (Figure 3D). Of the Ty1 or Ty2 elements that could be typed by established criteria [11] or mapped by a prior study [25], 21 are Ty1 and 5 are Ty2 (Figure S1D and Table 1; two could not be typed with available data). Since we did not determine the entire sequence of the Ty elements, it is unknown which are capable of autonomous transposition. No Ty4 element was found and none of the SGRP or NKY291 reads matched Ty4 internal sequences, but Ty4-derived solo LTRs are present (data not shown). Thus the Ty4 family is extinct in SK1.

Most LTR-retrotransposons generate sequence duplication at the integration site [34]. Among SK1 Ty elements whose insertion sites were precisely mapped, >95% showed perfect target sequence duplication with a good match to the consensus for elements in S288C (Table 1 and Figure S1E).

Ty integration can be potentially deleterious, by inactivating or altering expression of neighboring genes [35], [36]. However, obviously deleterious insertions are relatively rare in S288C [11]. Selection may account for some of this pattern, but target site bias is also a major factor: ∼90% of Ty1–Ty4 insertion sites in S288C (including solo LTRs) are near RNA pol III-transcribed genes such as tRNAs [11], mediated by interaction of Ty integrase with factors required for RNA pol III transcription [35]. Similarly, most SK1 Ty1, Ty2, and Ty3 elements (26 of 29) are near tRNA genes (Table 1), and one of the exceptions (at *ura3*) was selected because it conferred a desirable phenotype.

### A Genome-Wide View of DSBs near Ty Elements

**Figure 4:**
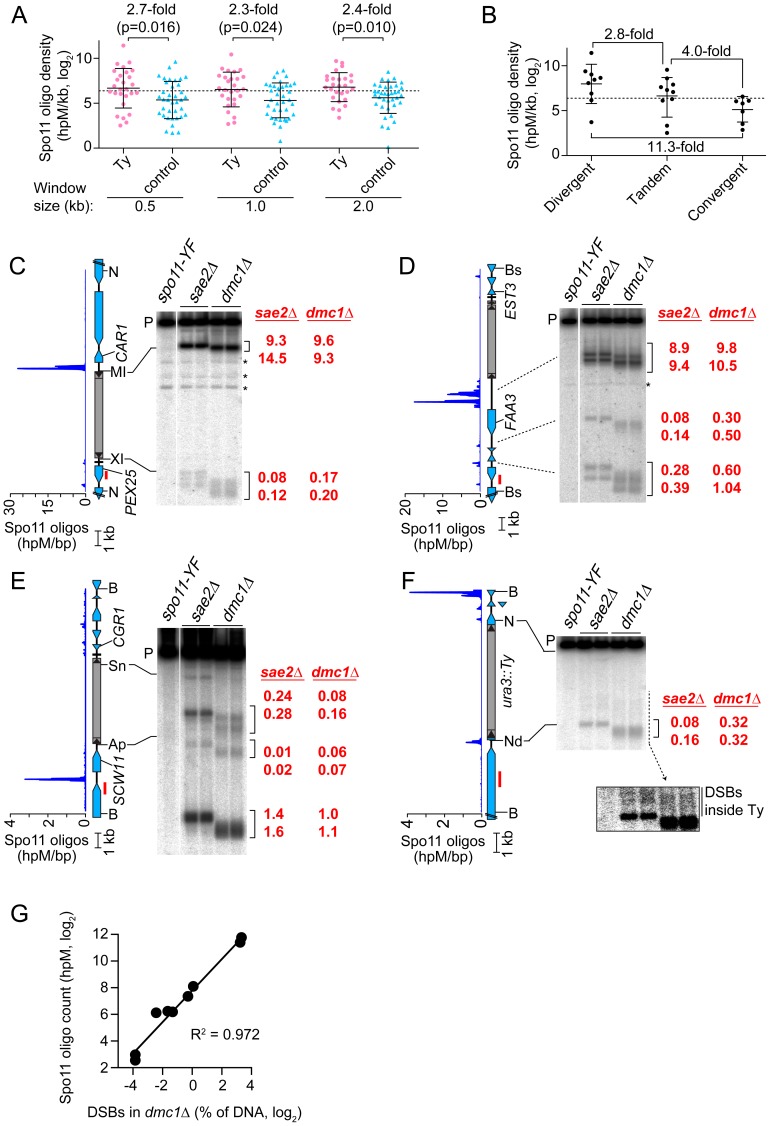
Meiotic DSBs in and around Ty elements. (A) Spo11 oligo densities around Ty elements. For each SK1 Ty, Spo11 oligo densities (hits per million mapped reads (hpM) per kb) were determined in the indicated window of adjacent sequence. Sites where Tys are present in S288C but absent in SK1 serve as controls. Bars are means and standard deviations; the dashed line is the genome average; p values are from Wilcoxon rank sum tests. For comparison, the internal Spo11 oligo density averaged across all Ty elements was 6.7 hpM/kb, approximately 30–40-fold lower than the mean for these flanking regions. (B) Spo11 oligo densities around Ty elements in different types of intergenic regions. (C–F) Physical detection of DSBs. (Left) Spo11 oligo distribution from Pan et al. (2011) and maps of ORFs (blue-filled polygons) and tRNA genes (horizontal bars). (Right) Southern blots of genomic DNA isolated from *spo11-Y135F*, *sae2Δ* and *dmc1Δ* strains at 6 hrs after entry into meiosis. Red numbers are DSB frequencies within the bracketed regions in each lane (% of total hybridization signal in the lane); quantification is provided separately for each lane, representing independent cultures. Red bars, probe positions; P, unbroken (parental) restriction fragments; asterisks, cross hybridizing bands. Flanking restriction sites plus internal sites used to generate genomic DNA markers (run on the same gels; not shown) are indicated: NcoI (N), BsaXI (XI), PpuMI (MI), Bsu36I (Bs), BglII (Bg), BspHI (HI), BamHI (B), ApaLI (Ap), SnaBI (Sn), NdeI (Nd). In (F), the inset shows a more exposed contrast of the phosphorimager signal for the region indicated by the dashed line. (G) Quantitative agreement between Spo11 oligo counts and DSB frequencies at hotspots near Ty elements in *dmc1Δ* mutants. DSB values are the means of the two independent cultures shown in panels C–F.

We previously showed that Spo11 oligo counts covary linearly with DSB levels, so the frequency of mapped Spo11 oligos is a proxy for DSB frequency [19]. To assess global trends for DSB formation near Ty elements, we compiled densities of Spo11 oligos within 0.5, 1, and 2 kb windows on both sides of each SK1 Ty (Figure 4A and Table S1). These densities varied widely between different Ty insertion sites, covering 80 to 500-fold ranges, depending on window size. Many Ty-flanking regions differed substantially from genome average, both hotter and colder. There was no obvious distinction between Ty families, in that the five elements unambiguously identified as Ty2 showed 33-fold variation in local Spo11 oligo density, and overlapped extensively with densities for Ty1 elements (p = 0.25, Wilcoxon rank sum test) (Table S1).

The mean Spo11 oligo density near Ty elements was higher than genome average, irrespective of window size (Figure 4A). However, since Tys are not randomly positioned, genome average may not be the most informative comparison. Although SK1 does not have full-length Ty elements where most of the Tys in S288C are found, integration bias with respect to tRNA genes was similar in the two strains. We reasoned that S288C integration sites can be viewed as potential integration sites in SK1, i.e., that S288C sites provide a good negative control for correlations between DSBs and Ty presence. In three window sizes analyzed, Spo11 oligo densities around these control sites varied as widely as for bona fide Ty integration sites (Figure 4A). However, while the density ranges overlapped, the values were consistently higher around SK1 Ty elements than around control sites, with mean Spo11 oligo densities 2.3–2.7-fold higher around the SK1 Tys (Figure 4A). We conclude that natural Ty insertion sites display a great degree of individual variability with respect to local Spo11 activity, comparable to the variability that would be seen for similar genomic locations without a Ty present. Moreover, these data do not provide evidence that Ty presence invariably causes DSB suppression nearby, and instead raise the possibility that Tys may tend to increase the local likelihood of DSB formation.

DSBs are preferentially formed at RNA pol II promoters [8], [37]. Intergenic regions between divergent transcription units, i.e., containing two promoters, tend to be somewhat hotter on average than intergenic regions between tandemly oriented genes, i.e., with just one promoter, while intergenic regions between convergent transcription units tend to be much colder than either [19]. All SK1 Ty elements, except the one in *ura3*, are in intergenic regions. When Ty elements were divided according to type of intergenic region, the local Spo11 oligo densities mirrored the trends seen for all intergenic regions genome-wide: Tys in divergent regions tended to have more Spo11 oligos mapped nearby than Tys in tandem regions, and both tended to be hotter than Tys in convergent regions (p = 0.0337, one-way ANOVA; Figure 4B). These findings imply that Ty elements do not necessarily override the intrinsic DSB-forming potential of the intergenic regions where they reside.

### Direct Analysis of DSBs near Ty Elements

Spo11 oligo patterns were confirmed by direct detection of DSBs near a subset of Ty elements. Since meiotic DSBs are transient in wild type, DSBs were detected in repair-deficient mutants. Sae2 is required for removal of Spo11 from DSB ends, so *sae2* mutants accumulate unresected DSBs that can be precisely mapped [2], [38]–[40]. However, these DSBs can differ quantitatively from wild type in a region-specific manner, for unknown reasons [41]. Dmc1 is a meiosis-specific strand exchange protein; *dmc1* mutants can remove Spo11 and generate ssDNA tails, but are unable to carry out further recombination steps and thus accumulate hyper-resected DSBs that migrate faster on agarose gels [42]. Wild-type DSB distributions appear to be more faithfully represented in *dmc1* mutants [41], [43]. Genomic DNA was purified from meiotic cultures of these mutants, restriction digested, and DSBs were detected by Southern blotting and indirect end-labeling (Figures 4C–4F). We chose four sites for physical analysis, reflecting a range of local Spo11 oligo distributions. As detailed below, all four showed good agreement between DSBs and Spo11 oligo maps, both quantitatively and spatially (Figures 4C–4G).

Ty*_PEX25-CAR1_* had the highest Spo11 oligo density nearby because of a strong hotspot immediately adjacent to its 5′ LTR (Figure 4C and Table S1). This hotspot was among the hottest 0.5% of all hotspots compiled previously [19]. A much weaker hotspot was also present adjacent to the 3′ LTR. Ty*_EST3-FAA3_* also had a strong hotspot near its 5′ LTR (Figure 4D). This hotspot was again within the hottest 0.5%, but was relatively wide. A weaker hotspot was present on the 3′ side of this Ty, close to a tRNA gene and the *EST3* promoter (discussed further below). Both Ty*_PEX25-CAR1_* and Ty*_EST3-FAA3_* are in intergenic regions containing a tRNA gene between divergently transcribed genes (Figures 4C and 4D). In both cases, the region next to the 5′ LTR carries the strong hotspot even though the region next to the 3′ LTR also contains a promoter. These two loci demonstrate that presence of a Ty can be compatible with very high DSB activity nearby.

Ty*_CGR1-SCW11_* showed weak DSB levels adjacent to the 5′ LTR (Figure 4E) as well as within the Ty, discussed below. This Ty is in an intergenic region containing a tRNA gene between convergent genes. A modest DSB and Spo11 oligo hotspot was also observed ∼2 kb away in the *SCW11* promoter (Figure 4E). Ty*_URA3_* also had a weak DSB hotspot nearby (Figure 4F). This hotspot was in the *ura3* promoter, coinciding with the 5′ LTR side of the Ty. Trace numbers of Spo11 oligos mapped in the *ura3* coding sequence adjacent to the 3′ LTR, but the corresponding DSB signal was too weak to be detected (Figure 4F and data not shown). Ty*_CGR1-SCW11_* and Ty*_URA3_* exemplify a situation in which presence of a Ty correlates with low DSB levels nearby, but do not speak to whether the Ty causes the low DSB activity.

### DSBs inside Ty Elements

Spo11 oligo mapping showed that meiotic DSBs occur within Ty elements [19], but individual Tys could not be evaluated. Physical analysis revealed a modest DSB hotspot inside Ty*_CGR1-SCW11_* (Figure 4E). DSBs overlapped the 5′ LTR and a region ∼1.8 kb from the 5′ end of the Ty, inside the Gag coding sequence. DSB signal was not detected near the 3′ end when the Southern blot was reprobed from the opposite side of the restriction fragment (data not shown), thus DSBs are more frequent near the 5′ end for this Ty. The Ty element that disrupts *ura3* also showed evidence of DSBs near its 5′ end, but at a level too low to be quantified (Figure 4F, inset). We did not observe discrete DSB signals inside either Ty*_PEX25-CAR1_* or Ty*_EST3-FAA3_* (Figures 4C and 4D), so these Tys lack hotspots above the limit of detection by Southern blotting (∼0.01% of DNA). Infrequent, relatively disperse DSBs would not be detected in this analysis. These results show that Tys differ significantly from one another in terms of number and location of internal DSBs. Interestingly, break levels in the flanking regions do not necessarily correlate with levels inside the Ty.

### Chromatin Structure in and near Tys

**Figure 5:**
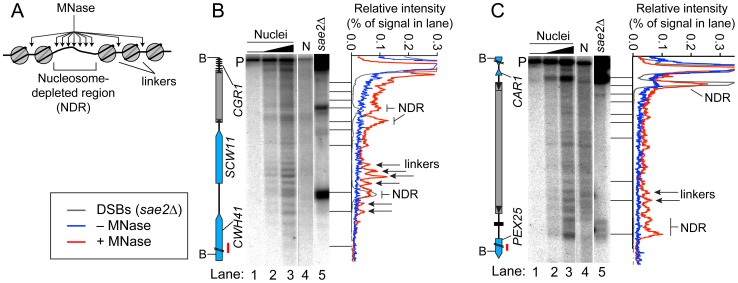
Chromatin structures of Ty elements. (A) Preferential MNase cleavage of chromatin in nucleosome-depleted regions (NDR) and linkers between nucleosomes. (B–C) MNase sensitivity of regions in and around Ty*_CGR1-SCW11_* (B) and Ty*_PEX25-CAR1_* (C). Intact meiotic nuclei were treated with 0, 2.5×10^−5^, or 5×10^−5^ units of MNase per µg of DNA (lanes 1–3) and purified genomic DNA (N, for naked DNA) from vegetative cells was treated with 1.6×10^−4^ units per µg DNA (lane 4), then MNase cleavage patterns were determined by Southern blotting and indirect end-labeling. Genomic DNA prepared from meiotic *sae2Δ* cells is a marker for DSB positions (lane 5). Profiles of lanes 1 (−MNase), 3 (+MNase), and 5 (DSBs) are shown to the right of each blot. Red bars on ORF maps indicate probe positions.

Open chromatin structure provides a window of opportunity for Spo11-dependent DSB formation [37]. To investigate the relationship between DSBs and chromatin structure at Ty elements, intact nuclei were prepared from meiotic cultures of wild-type cells and partially digested with micrococcal nuclease (MNase). DNA was extracted and digested with appropriate restriction enzymes, and MNase cleavage sites were identified by Southern blotting and indirect end-labeling (Figure 5). MNase digestion of purified genomic DNA was examined in parallel. Nucleosomal DNA is relatively resistant to MNase cleavage (Figure 5A). For example, the *SCW11* promoter showed a broad band of preferred MNase digestion indicative of a nucleosome-depleted region (NDR) typical of many yeast promoters, flanked by ladders of bands from cleavage in the linkers between positioned nucleosomes upstream and downstream of the promoter (Figure 5B, lanes 2–3). As expected, the DSB hotspot in the *SCW11* promoter corresponded to the MNase-hypersensitive NDR (Figure 5B, lanes 2–3 vs. lane 5).

Ty*_CGR1-SCW11_* showed dispersed MNase cleavage inside, with two prominent MNase-hypersensitive zones toward its 5′ end, one of which corresponded to the DSB hotspot within this Ty (Figure 5B, lanes 2–3 vs. 5). Within each hypersensitive zone a weak banding pattern could be seen, suggesting a modest tendency for nucleosomes to occupy certain preferred positions in subpopulations of cells. Within the Ty element, 28.3% of DNA was cleaved (4.7% per kb), compared with 30.7% of DNA cleaved between the 5′ LTR and the end of *CWH41* (11.8% per kb). Thus, this Ty overall is only about two-fold more resistant to MNase than the intergenic and genic regions flanking it.

In contrast, Ty*_PEX25-CAR1_* appeared less sensitive to MNase compared to flanking genic regions. Whereas 17.3% of DNA was cleaved in the intergenic region between the 3′ LTR and the start of *PEX25* (43% per kb), 33.3% of DNA was cleaved within Ty*_PEX25-CAR1_* (5.6% per kb). Ty*_PEX25-CAR1_* did not show prominent hypersensitivity toward its 5′ end (Figure 5C, lanes 2–3). Instead, it showed a broad region of modest hypersensitivity at its 3′ end, suggestive of an array of weakly positioned nucleosomes extending into the flanking intergenic region. These results show that chromatin structure can vary between individual Ty elements. Importantly, MNase-hypersensitive sites indicative of NDRs were present at both the strong DSB hotspot in the *CAR1* promoter and the weaker hotspot in the *PEX25* promoter flanking Ty*_PEX25-CAR1_* (Figure 5C, lanes 2–3 vs. 5). Thus, presence of a Ty close by need not result in elimination of the open chromatin structure typical of promoters and DSB hotspots.

### Ty Elements Can Stimulate DSB Formation Nearby

**Figure 6:**
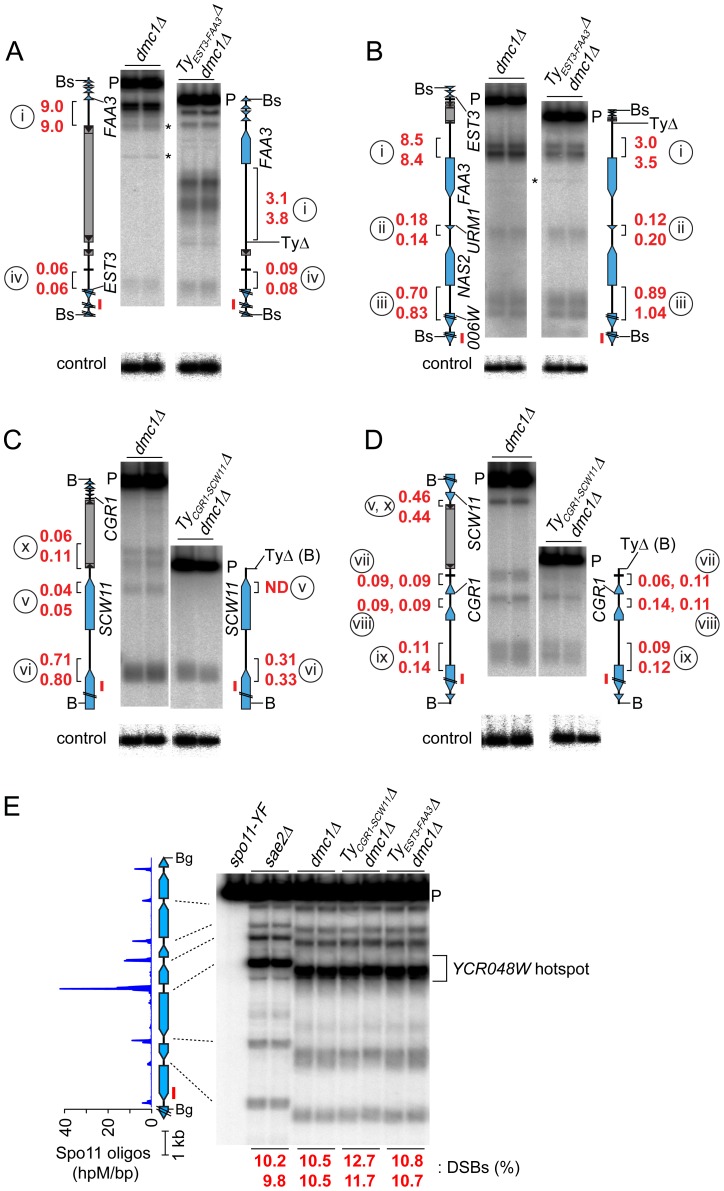
Deleting Ty elements increases DSB formation nearby. (A–D) Genomic DNA was isolated from meiotic cultures of a *dmc1Δ* strain containing the full complement of SK1 Tys and *dmc1Δ* strains in which either Ty*_EST3-FAA3_* or Ty*_CGR1-SCW11_* was deleted. DSBs were detected by Southern blotting and indirect end-labeling. Figures are labeled as in Figures 4C–F. Circled lower case roman numerals indicate hotspots discussed in the text. Red numerals are DSB frequencies within the bracketed regions in each of two independent cultures, corrected where appropriate for differences in transfer efficiency for the parental fragments (see Materials and Methods). Blots were stripped and rehybridized to probes from separate loci to serve as loading controls (lower panels). (A,B) DSBs around the Ty*_EST3-FAA3_* insertion site, probed from either side. (C,D) DSBs around the Ty*_CGR1-SCW11_* insertion site, probed from either side. (E) DSBs at the *YCR048W* hotspot (control locus) in the same samples as in panels A–D.

To test whether natural Ty elements directly affect adjacent DSB formation, we individually deleted two Tys and compared DSB patterns with and without these elements present. As a control, we quantified DSBs in the same cultures at the *YCR048W* hotspot on Chr III; DSBs at this hotspot were similar between the parental and Ty deletion strains (Figure 6E).

Remarkably, a strain lacking Ty*_EST3-FAA3_* experienced ∼2–3 fold fewer DSBs in the *FAA3* promoter region than the parental strain carrying this Ty (hotspot i in Figures 6A and B). Results were similar irrespective of which side of the genomic restriction fragment was probed. Although DSB levels were different, their distribution within the hotspot was unchanged (Figure 6B). The other hotspots in the probed region were affected little if at all in the strain lacking the Ty (hotspots ii, iii, and iv in Figures 6A and 6B).

In a strain lacking Ty*_CGR1-SCW11_*, the weak DSB signal near the 5′ end of the Ty element became undetectable (hotspot v, Figure 6C), and the hotspot in the *SCW11* promoter showed 2.3-fold lower DSBs than the parental strain (hotspot vi, Figure 6C). The weak hotspots on the other side of the Ty insertion site were essentially unchanged (hotspots vii–ix, Figure 6D). As expected, the DSB signal inside the retrotransposon was not observed in the Ty-deletion strain (hotspot x, Figure 6C), but no new DSB signal arose in its place as would have been expected if presence of the Ty were suppressing an otherwise active DSB site.

These findings do not support the hypothesis that Ty elements invariably suppress meiotic DSB formation in their vicinity. Instead, we conclude that at least some Ty insertions cause an increase in DSBs nearby.

## Discussion

Prior analyses of nucleotide variation demonstrated that SK1 is genetically distant from S288C [26], [44]. Accordingly, we find that the catalogs of full-length Ty elements are completely different in these strains, except for an ancient and immobile copy of Ty5. Full-length Tys are prone to loss by LTR-LTR recombination [45]. S288C does not have full-length Tys or solo LTRs where Ty elements reside in SK1 (data not shown), suggesting that transposition of the SK1 Tys occurred after SK1 and S288C diverged from their last common ancestor. While SK1 does not have full-length Tys at the same sites as in S288C, we did not comprehensively map solo LTRs, so it is possible that some S288C Ty elements predate divergence of the strains and were lost in SK1 by LTR-LTR recombination. It will be interesting to identify if any solo LTRs are shared between SK1 and S288C. Such LTRs would be “fossils” of ancestral transposition events, and comparison of their features with those of younger LTRs or Tys may illuminate how host-Ty element relationships have evolved.

In principle, the deep sequencing approach we used for Ty mapping should be broadly applicable to repetitive elements of any type in any organism. Indeed, while this work was in progress, others independently used a similar method to identify new transposon insertions in *Drosophila* [46]. This approach, combined with growing libraries of whole-genome, paired-end sequencing data from widely divergent *S. cerevisiae* strains, will facilitate assembly of complete genome sequences and also permit genealogical analysis of Ty insertion site diversity.

Chromosomal rearrangements can arise in vegetatively growing cells as a consequence of NAHR between Ty elements [12]–[15], [47]–[49]. Ty location and orientation dictate the degree of susceptibility to rearrangement, the structures of rearranged chromosomes, and whether the outcome is deleterious, neutral, or advantageous. For example, in S288C, deletion of *HTA1-HTB1* (one of two gene pairs encoding histones H2A and H2B) causes pleiotropic defects that both promote and select for amplification of the separate *HTA2-HTB2* locus [47]. Amplification occurs via NAHR between two flanking Ty elements in direct repeat orientation near the centromere of Chr II (see Figure 1). These Ty elements are not present in the W303 strain, so facile amplification of *HTA2-HTB2* is not possible and deletion of *HTA1-HTB1* is lethal in this strain [47]. SK1 also lacks similarly positioned Tys (Figure 1), so we anticipate that deletion of *HTA1-HTB1* would be lethal in this strain too. Moreover, closely juxtaposed Ty elements in inverted orientation can create fragile sites predisposed to chromosome rearrangement [14], [50]. SK1 has no instances of closely spaced, inverted, full-length Ty pairs, but the Ty fragment *Ty_EXG2-YDR262W-2_* is juxtaposed in inverted orientation to full-length *Ty_EXG2-YDR262W-1_* on Chr IV, and *Ty_NCE103-YNL035C-2_* is inserted in inverted orientation into *Ty_NCE103-YNL035C-1_* on Chr XIV. These are thus candidates for fragile sites in this strain. More generally, these scenarios (histone gene amplification and Ty-associated fragile sites) illustrate the importance of Ty maps in different strains because the particular details of Ty element distribution are critical for understanding the influence of these retrotransposons on genome instability and evolution of genome structure.

Ty-mediated NAHR also occurs during meiosis [4], [22], [23]. We show here that four individual Ty elements experience different frequencies of DSBs inside. To our knowledge, this is the first direct detection of meiotic DSBs in Ty elements, confirming the inference from Spo11 oligo mapping that significant numbers of DSBs occur within Tys [19]. We detected a total internal DSB frequency of at least 0.1–0.3% of DNA in *Ty_CGR1-SCW11_*. Assuming at most one DSB per four chromatids in a given cell, this frequency predicts that 0.4–1.2% of meiotic cells experience a DSB within this Ty element alone. This number is small on a per-cell basis, but becomes substantial when considered from the perspective of a population of cells or over many generations. Furthermore, we previously showed that ∼0.28% of Spo11 oligos map to Ty-derived sequences, indicating that one in every 2–3 meiotic cells experiences a DSB in a Ty or solo LTR, assuming an average of ∼160 DSBs per cell [19]. Excluding LTRs, ∼0.1% of Spo11 oligos map to Ty-internal sequences, which predicts a DSB frequency of 1.1–2.5% of DNA summed over all Ty elements, based on linear regression of Spo11 oligo counts vs. DSB levels (see Materials and Methods). This estimate is higher than the total DSB frequency observed in the four Tys assayed here, so it is likely that other Ty elements experience a significant number of DSBs as well.

Based on copy number compiled here, we estimate that Tys account for ∼1.5% of genomic DNA, not including rDNA or the contribution of solo LTRs. In turn, this suggests that DSBs within Tys are ∼15-fold suppressed relative to genome average since only ∼0.1% of total Spo11 oligos came from Ty-internal sequences. However, genome average includes many strong DSB sites, such as promoters, that are structurally and functionally dissimilar from the inside of a Ty, which is principally coding sequence. Genome wide, coding sequences account for only ∼11.5% of Spo11 oligos but occupy ∼69.4% of the genome. Thus, on average, Tys are only ∼2–3-fold colder than the typical open reading frame.

Our findings have implications for understanding behavior of outcrossed yeast strains: as a consequence of different Ty distributions, any DSB within a Ty would lack a recombination partner at the allelic position, so such DSBs are most likely repaired from the sister chromatid, by NAHR, or by single-strand annealing between 5′ and 3′ LTRs (which deletes the Ty-internal sequence leaving behind a solo LTR). It will be interesting to determine whether large-scale differences in Ty distributions contribute to reduced ability of hybrids to produce viable spores [51], [52], in turn contributing to reproductive barriers between strains. Our findings also have implications for inbred strains, including diploids produced by homothallic strains: DSBs within Tys have potential to provoke NAHR even if there is a Ty present at the allelic position on the homologous chromosome. Such NAHR may contribute to sequence homogenization and co-evolution of Ty elements. Moreover, our results provide a framework for studying mechanisms that act after DSB formation to minimize the risk of deleterious chromosome rearrangements [6].

Chromatin structure may play an important role in DSB formation within Ty elements, as suggested by the observation of MNase hypersensitivity at the 5′ end of Ty*_CGR1-SCW11_* where DSBs are formed. Our findings show that different Tys can have different chromatin architecture. In a similar vein, relative transcription levels of Ty1 elements in vegetatively growing S288C were found to differ by ∼50 fold [24]. Thus, Ty elements can differ greatly from one another, precluding generalization of a one-size-fits-all pattern from any given element.

Although DSBs wholly within Tys have greater potential to instigate NAHR, breaks in unique sequences near Ty elements may also be at risk because DSB resection generates recombinogenic ssDNA for significant distances (up to a kb or more) from the Spo11 cleavage site [53]–[55]. We find here that DSB levels vary substantially in regions flanking different Ty elements and that presence of a Ty does not invariably cause suppression of adjacent DSB activity. These findings are counter to predictions from prior analysis of a Ty in the *HIS4* promoter [16], further highlighting the individual variability of Ty elements.

We propose that differences between the studies reflect aspects of host-transposon interactions that evolved to minimize deleterious effects of retrotransposition. The Ty at *HIS4* mimicked a spontaneous Ty integration that disrupted *HIS4* expression (*Ty917*) [20], [22]. It was inserted ∼70 bp upstream of *HIS4*, moving the TATA box and upstream activator sequence ∼6 kb away from their normal position and eliminating the DNase I hypersensitivity of the *HIS4* promoter [16]. The altered chromatin structure was interpreted as spreading of closed chromatin from the Ty into surrounding regions [16], but an alternative interpretation is that *Ty917* is simply an insertional mutation that compromises the cis-acting elements defining the *HIS4* promoter NDR, thereby disrupting both promoter activity and Spo11 access. In this view, the effect of *Ty917* on DSB formation is context dependent and intimately tied to its deleterious effect on a host gene.

In contrast to *Ty917*, most naturally occurring Ty elements are found near tRNA or other RNA pol III-transcribed genes, likely targeted there via interactions of integration complexes with RNA pol III transcription machinery [11], [56], [57]. Ty elements inserted near (and especially upstream of) tRNA genes will tend to be distant from regulatory regions of other adjacent genes because the mean distance between tRNA genes and their upstream neighbors (excluding Ty and LTR sequences) is ∼500 bp larger than the distance from tRNAs to downstream genes or the average size of intergenic regions genome-wide [58]. Thus, while Ty integration site preference may have evolved to prevent deleterious mutations [11], [36], [59], it has the additional consequence that Ty elements tend to avoid the very RNA pol II promoters where most meiotic DSBs are formed, and tend not to impinge on promoter properties that favor Spo11 activity, such as transcription factor binding and nucleosome depletion. Our direct analysis of chromatin structure and DSB formation around *Ty_PEX25-CAR1_* supports this view. The correlation between DSB levels and the class of Ty-bearing intergenic region (Figure 4B) also supports this idea by implying that DSB frequency is substantially influenced by the local DSB-forming potential of the neighborhoods where Ty elements reside.

We were surprised to find that deletion of two Ty elements in different genomic contexts caused decreased DSB formation nearby. Thus, at least some Tys stimulate adjacent DSB formation, and our genome-wide analysis suggested this may be a fairly general property. The mechanism behind this effect is as yet unclear. Both Ty deletions showed an apparent polarity in that the regions where DSB levels were most affected were adjacent to the 5′ LTRs. Although sample size is too small to know if this is a general pattern, it may indicate that adjacent DSB formation is modulated by properties of Ty 5′ LTRs, which in some cases carry promoter activity and contain binding sites of transcriptional activators [e.g., 24]. Alternatively, it may be that DSB stimulation is not a unique property of the Ty itself, but instead is simply a consequence of a structural change in the chromosome. Indeed, there are numerous examples where heterologous DNA insertions generate new DSB hotspots [reviewed in 8], although such insertions rarely, if ever, cause enhanced activity of natural, promoter-associated hotspots nearby.

Regardless of the mechanism, this finding has implications for inheritance of Tys across sexual cycles. The chromosome that experiences a DSB is the recipient of genetic information from its homologous partner, in part because of net degradation of the broken chromosome by DSB resection and resynthesis using the intact partner as the template [60]. As a consequence of this gene conversion bias, an allele with a higher propensity toward DSB formation will tend to be under-transmitted during meiosis. Thus, elevated DSB frequency near Tys might tend to favor elimination of Ty copies by meiotic recombination in diploids heterozygous for the Ty insertion. In principle, this tendency could affect new Ty insertions in a diploid or inbred population, as well as older Ty insertions in outcrosses between diverged strains. Our findings thus raise new questions about retrotransposon-host relationships and the roles of the intersection between Ty elements, meiotic recombination initiation, and NAHR.

## Materials and Methods

### Yeast Strains and Sequencing

Yeast strains are listed in Table S2. Ty elements were deleted by two-step gene replacement, resulting in precise replacement of each Ty element with a diagnostic restriction site (see legend to Tables S2 and S3). Other alleles were introduced by genetic crosses or by one-step gene replacements using standard methods. All gene replacements were confirmed by Southern blotting. A whole-genome mate pair library was prepared according to manufacturer's recommendations (Roche) from genomic DNA purified from a vegetative culture of NKY291, and sequenced on the Roche 454 platform. The NKY291 library had an average sequence length of 173 bp and an average insert size of 2.8 kb (16-fold coverage in 653,261 sequence pairs). Sequence data are available at http://cbio.mskcc.org/public/SocciN/SK1_MvO/Data/GCL0188__454__PE_3k/

### Identification of Ty Elements in SK1

To evaluate presence of Tys from S288C or previously identified in SK1 [25], SK1-derived sequence reads from the SGRP were viewed in the genome browser provided by the Sanger Institute (http://www.sanger.ac.uk/research/projects/genomeinformatics/sgrp.html). When read alignment patterns were indicative of Ty presence, partial DNA sequences of the Tys were deduced from contigs assembled from these reads and used to determine Ty orientation and family, by comparison to exemplars of Ty families from S288C. Ty insertion sites were mapped by identifying SGRP reads overlapping boundaries between Tys and flanking genomic sequence. If no overlapping reads were present, PCR products spanning the Ty-element-containing region were partially sequenced to determine the precise insertion sites.

For systematic Ty mapping, the SK1 mate pair libraries from SGRP and from our sequencing of NKY291 were mapped against a compilation of non-LTR portions of S288C Ty elements. Mapping was performed using LastZ on the Galaxy server (http://main.g2.bx.psu.edu/). Mate pairs of reads that aligned with Ty-internal sequence were then mapped onto the S288C genome using LastZ, and reads that mapped to multiple positions were discarded. Candidate Ty insertion sites identified from the remaining reads were validated by manual inspection of sequence alignments and/or PCR of genomic DNA. In addition to the full-length Tys and large Ty fragments listed in Table 1, this analysis identified three small (∼120–180 bp) non-LTR Ty fragments at ∼805 kb on Chr IV, ∼78 kb on Chr VIII, and ∼338 kb on Chr XVI (data not shown). How these insertions arose is uncertain, but because they are so short, they were not considered as Tys in this study.

### Meiotic Culture and Physical DSB Detection

Synchronous meiotic cultures were prepared essentially as described [61]. Cells were harvested from a single culture of SKY4121, two independent cultures of SKY4151 and of SKY4153, and single cultures of SKY4188, SKY4189, SKY4191 and SKY4192 at 6 hr in meiosis, and genomic DNA was isolated in low melting temperature agarose plugs, digested with appropriate restriction enzymes, electrophoresed on agarose gels, and analyzed by Southern blotting and indirect end-labeling, as described previously [19], [61]. Restriction enzymes and probes are as follows and primers used to prepare probes are in Table S3: Ty*_PEX25-CAR1_*, *BamH*I, *PEX25* probe; Ty*_EST3-FAA3_*, *Bsu*36I, *DOT5* or *EPS1* probe; Ty*_CGR1-SCW11_*, *BamH*I, *RPS24A* or *CWH41* probe; Ty*_URA3_*, *BamH*I, *GEA2* probe; *YCR048W* hotspot, *Bgl*II, *RCS6* probe. Hybridization signal was detected and quantified with Fuji phosphor screens and ImageGauge software. DSB frequency was determined as the percent of radioactivity in DSB fragments relative to total radioactivity in the lane. Signals from the *spo11-Y135F* strain were used to subtract background.

The large difference in size of the parental-length restriction fragments between Ty-containing and Ty-deleted loci (experiments in Figures 6A–6D) could be expected to cause differences in Southern blot transfer efficiencies, which could lead to incorrect estimates of relative DSB levels. To account for this, we applied the following strategy. First, Ty+ and TyΔ samples were run on the same gel, transferred together, and hybridized together to the appropriate probe for the Ty locus. The membranes were then stripped and re-hybridized to probes from different loci to serve as loading controls: *YCR057C* probe for the blots shown in Figures 6A and 6B and *YKL182W* probe for the blots in Figures 6C and 6D (Table S3). We used the loading controls to correct DSB estimates by assuming that there was “missing signal” from the Ty+ lanes because of less efficient transfer of Ty-containing DNA fragments. From this analysis, we estimated that the parental bands in the Ty-containing strains were transferred at ≥75% the efficiency seen with the Ty-deletion strains (data not shown).

### MNase Digestion of Chromatin

Meiotic culture of wild-type diploid, SKY41, was prepared as described above. Intact meiotic nuclei were prepared 4 hrs after induction of sporulation by spheroplasting, hypotonic lysis, and centrifugation on sucrose step gradients, as described previously [62]. Nuclei were quantified by fluorometry with Hoechst 33258 dye. A volume of nuclear suspension containing 4 µg DNA was diluted with an equal volume of ice-cold 10 mM Tris-HCl, pH 8.0, 5 mM MgCl_2_ and 1 mM Pefabloc. Nuclei were collected by centrifugation and resuspended in 90 µl of 10 mM Tris-HCl, pH 8.0, 2.5 mM CaCl_2_, 3.5 mM MgCl_2_ on ice. Ten µl of appropriate concentration of MNase (Worthington) was added, digestion was performed for 5 min at 37°C, then terminated by addition of 0.4 ml of 62.5 mM EDTA, 125 mM Tris-HCl, pH 8.0, 0.625% SDS and 5 µl of 20 mg/ml proteinase K. Samples were incubated at 58°C for 2 hrs to overnight. DNA was extracted twice with phenol∶chloroform∶isoamyl alcohol (25∶24∶1) and once with chloroform, then precipitated with isopropanol with 10 µg of glycogen and dissolved in 10–20 µl of dH_2_O. As a control, genomic DNA was purified from vegetatively growing cells and treated with MNase, followed by purification as above. DNA from MNase-treated nuclei or naked DNA was digested with *BamH*I, electrophoresed on agarose gels, and analyzed by Southern blotting and indirect end-labeling using the *CWH41* probe (Ty*_CGR1-SCW11_*) or *PEX25* probe (Ty*_PEX25-CAR1_*) (Table S3).

### Analysis of Genome-Wide DSB Mapping Data

For the analysis in Figure 4A, groups of closely neighboring Tys in SK1 (between *EXG2* and *YDR262W* on Chr IV, and between *NCE103* and *YNL035C* on Chr XIV) were treated as single Tys. Furthermore, the Ty5 at the left end of Chr III was excluded, as DSBs are known to be suppressed in subtelomeric regions [19], [41], [43]. Therefore, Spo11 oligo counts were determined in 27 Ty-bearing regions in SK1 (Table S1). As controls, we used the coordinates of S288C Ty elements. We excluded S288C Ty positions within 2 kb of SK1 Ty elements, closely neighboring Tys were considered as a single element, and *YCLWTy5-1* was excluded as above. Spo11 oligo densities adjacent to 37 control sites were determined.

To estimate the genome-wide percentage of DNA broken in Tys, we summed Spo11 oligos that mapped to non-LTR Ty sequences. Excluding LTRs means that we are underestimating DSBs associated with full-length Tys, but this is necessary because we cannot distinguish Spo11 oligos from LTRs flanking Ty elements from those originating within solo LTRs or LTR fragments. Using our previously defined regression relationship [19], we converted Spo11 oligo counts to DSB frequency, yielding estimates in *dmc1* and *sae2* background of 2.0% and 0.85%, respectively. Since the prior study used the *spo11-HA* strain, which forms DSBs at a reduced frequency of ∼80% of a *SPO11*+ strain [63], we therefore estimate the Ty DSB frequency to be ∼2.5% in *dmc1* and 1.1% in *sae2* in the *SPO11+* background.

## Supporting Information

Figure S1 Ty elements in SK1. (A,B) SK1 sequence reads mapped to the S288C genome near positions of S288C Ty elements. The color scheme is as in Figure 2. The vertical pink arrow indicates an SK1-specific Ty. In (A), the Ty site was spanned by read pairs with large apparent inserts (>8 kb) and no reads mapped across the predicted boundaries between Ty and adjacent sequence, indicating that this Ty does not exist in SK1. In (B), SGRP data again showed that SK1 lacks a Ty(s) at the precise position, but orphan reads pointed to a nearby insertion relative to S288C. Presence of a Ty was subsequently confirmed by PCR of genomic DNA (data not shown). (C) SK1 sequence reads near a region (black bar) previously identified as Ty-containing in SK1 [25]. where SK1 Ty1 or Ty2 were mapped. Orphans in the SGRP read map revealed an insert in SK1, and analysis of the mate pairs of the orphans showed that the inserts contain Ty sequences. PCR and sequencing of genomic DNA confirmed the presence of an SK1-specific Ty (data not shown). (D) Ty1 and Ty2 family members in SK1. LTR sequences of 24 Ty elements were aligned with the LTR of Ty1-H3, a Ty1 element identified in strain JB84A [64], by Clustal W using the MegAlign program (DNASTAR). LTRs of Ty1 and Ty2 families were distinguished by the presence or absence of the T residue indicated in red, which corresponds to base 284 of the Ty1-H3 LTR. (E) Target site consensus sequence of SK1 and S288C retrotransposons. The SK1 consensus sequence was derived from the 5-bp duplications at insertion sites of 23 Ty elements. The S288C consensus sequence is from 118 Ty and LTR insertion sites with a 5-bp duplication [11].
(PDF)

Table S1 DSB activities in the 0.5-kb regions flanking Ty elements.
(PDF)

Table S2 *S. cerevisiae* strains used in this study.
(PDF)

Table S3 Primers used in this study.
(PDF)
