# Supplementary figures and images for: Meiotic Recombination Initiation in and around Retrotransposable Elements in Saccharomyces cerevisiae
Source: PLoS Genet. 2013 Aug 29;9(8):e1003732. doi: 10.1371/journal.pgen.1003732 (PMC3757047; doi:10.1371/journal.pgen.1003732)

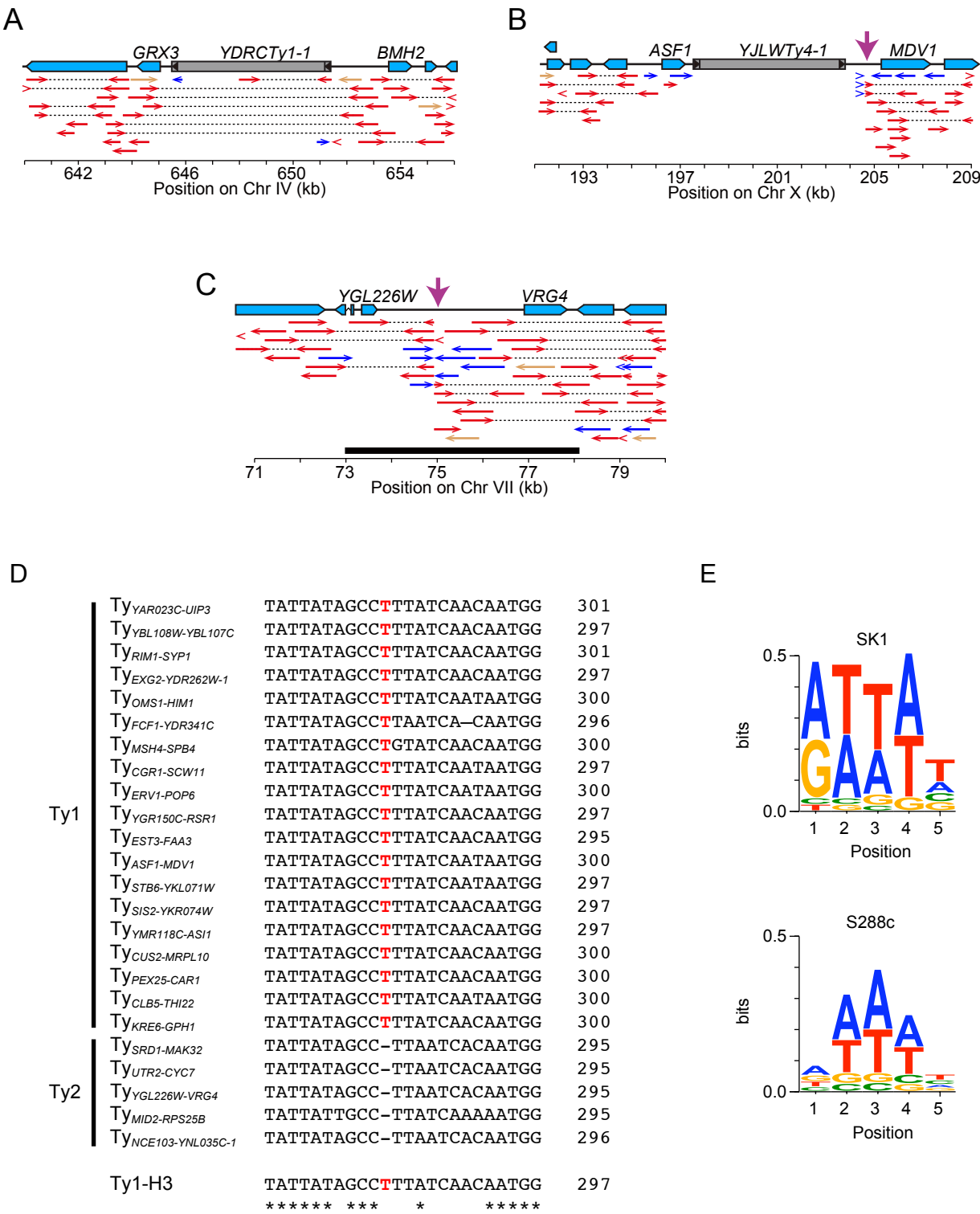

Supplement: Figure S1 — Ty elements in SK1. (A,B) SK1 sequence reads mapped to the S288C genome near positions of S288C Ty elements. The color scheme is as in Figure 2. The vertical pink arrow indicates an SK1-specific Ty. In (A), the Ty site was spanned by read pairs with large apparent inserts (>8 kb) and no reads mapped across the predicted boundaries between Ty and adjacent sequence, indicating that this Ty does not exist in SK1. In (B), SGRP data again showed that SK1 lacks a Ty(s) at the precise position, but orphan reads pointed to a nearby insertion relative to S288C. Presence of a Ty was subsequently confirmed by PCR of genomic DNA (data not shown). (C) SK1 sequence reads near a region (black bar) previously identified as Ty-containing in SK1 [25]. where SK1 Ty1 or Ty2 were mapped. Orphans in the SGRP read map revealed an insert in SK1, and analysis of the mate pairs of the orphans showed that the inserts contain Ty sequences. PCR and sequencing of genomic DNA confirmed the presence of an SK1-specific Ty (data not shown). (D) Ty1 and Ty2 family members in SK1. LTR sequences of 24 Ty elements were aligned with the LTR of Ty1-H3, a Ty1 element identified in strain JB84A [64], by Clustal W using the MegAlign program (DNASTAR). LTRs of Ty1 and Ty2 families were distinguished by the presence or absence of the T residue indicated in red, which corresponds to base 284 of the Ty1-H3 LTR. (E) Target site consensus sequence of SK1 and S288C retrotransposons. The SK1 consensus sequence was derived from the 5-bp duplications at insertion sites of 23 Ty elements. The S288C consensus sequence is from 118 Ty and LTR insertion sites with a 5-bp duplication [11]. (PDF) [file pgen.1003732.s001.pdf]
