# Supplementary material for: Meiotic Recombination Initiation in and around Retrotransposable Elements in Saccharomyces cerevisiae
Source: PLoS Genet. 2013 Aug 29;9(8):e1003732. doi: 10.1371/journal.pgen.1003732 (PMC3757047; doi:10.1371/journal.pgen.1003732)
Supplement: Table S1 — DSB activities in the 0.5-kb regions flanking Ty elements. (PDF) [file pgen.1003732.s002.pdf]

Table S1. DSB activities in the 0.5-kb regions flanking Ty elements

Rank	Name	Spo11 oligos (hpM/kb)	Chr	Ty family	Intergenic regions
1	$Ty_{PEX25\text{-}CAR1}$	2742.3	XVI	Ty1	Divergent
2	$Ty_{MSH4\text{-}SPB4}$	748.3	VI	Ty1	Tandem
3	$Ty_{SIS2\text{-}YKR074W}$	665.3	XI	Ty1	Divergent
4	$Ty_{CLB5\text{-}THI22}$	518.3	XVI	Ty1	Divergent
5	$Ty_{YMR118C\text{-}ASI1}$	405.6	XIII	Ty1	Divergent
6	$Ty_{FCF1\text{-}YDR341C}$	386.3	IV	Ty1	Tandem
7	$Ty_{EXG2\text{-}YDR262W\text{-}1}$	346.3	IV	Ty1	Divergent
	$Ty_{EXG2\text{-}YDR262W\text{-}2}$			Ty1 or Ty2	
8	$Ty_{YAR023C\text{-}UIP3}$	268.7	I	Ty1	Divergent
9	$Ty_{YGL226W\text{-}VRG4}$	240.8	VII	Ty2	Tandem
10	$Ty_{YGR150C\text{-}RSR1}$	197.9	VII	Ty1	Tandem
11	$Ty_{RIM1\text{-}SYP1}$	158.2	III	Ty1	Tandem
12	$Ty_{ASF1\text{-}MDV1}$	134.1	X	Ty1	Tandem
13	$Ty_{SRD1\text{-}MAK32}$	119.9	III	Ty2	Divergent
14	$Ty_{CUS2\text{-}MRPL10}$	95.6	XIV	Ty1	Convergent
15	Ty_{URA3}	92.9	V	Ty1	ORF
16	$Ty_{KRE6\text{-}GPH1}$	78.5	XVI	Ty1	Tandem
17	$Ty_{EST3\text{-}FAA3}$	71.0	IX	Ty1	Divergent
18	$Ty_{OMS1\text{-}HIM1}$	69.7	IV	Ty1	Tandem
19	$Ty_{NCE103\text{-}YNL035C\text{-}1}$	68.7	XIV	Ty2	Convergent
	$Ty_{NCE103\text{-}YNL035C\text{-}2}$			Ty1 or Ty2	
20	$Ty_{MID2\text{-}RPS25B}$	65.7	XII	Ty2	Convergent
21	$Ty_{ERV1\text{-}POP6}$	55.8	VII	Ty1	Convergent
22	$Ty_{YBL108W\text{-}YBL107C}$	29.9	II	Ty1	Tandem
23	$Ty_{YNL035C\text{-}YNL034W}$	13.2	XIV	Ty3	Divergent
24	$Ty_{CGR1\text{-}SCW11}$	11.4	VII	Ty1	Convergent
25	$Ty_{STB6\text{-}YKL071W}$	10.0	XI	Ty1	Tandem
26	$Ty_{UTR2\text{-}CYC7}$	7.2	V	Ty2	Convergent
27	$Ty_{YER137C\text{-}RTR1}$	5.8	V	Ty1	Tandem

The genome average Spo11 oligo density is 83.0 hpM/kb.
