# Supplementary material for: Meiotic Recombination Initiation in and around Retrotransposable Elements in Saccharomyces cerevisiae
Source: PLoS Genet. 2013 Aug 29;9(8):e1003732. doi: 10.1371/journal.pgen.1003732 (PMC3757047; doi:10.1371/journal.pgen.1003732)
Supplement: Table S2 — S. cerevisiae strains used in this study. (PDF) [file pgen.1003732.s003.pdf]

Table S2. *S. cerevisiae* strains used in this study

Name	Genotype
BY4741	*MATa, his3Δ1, leu2Δ0, met15Δ0, ura3Δ0*
NKY291	*MATa, ho::LYS2, lys2, leu2::hisG, ura3*
SKY41	*MATa/α, ho::LYS2/", lys2/", leu2::hisG/", nuc1Δ::LEU2/", arg4-nsp/", his4X::LEU2/", ura3/"*
SKY4016	*HO, LYS2, URA3, LEU2*
SKY4121	Same as SKY41, but *spo11-Y135F::kanMX/"*
SKY4151	Same as SKY41, but *dmc1Δ::hphMX/"*
SKY4153	Same as SKY41, but *sae2Δ::kanMX/"*
SKY4188	Same as SKY4151, but $Ty_{CGR1\text{-}SCW11}$Δ/"
SKY4189	Same as SKY4151, but $Ty_{CGR1\text{-}SCW11}$Δ/"
SKY4191	Same as SKY4151, but $Ty_{EST3\text{-}FAA3}$Δ/"
SKY4192	Same as SKY4151, but $Ty_{EST3\text{-}FAA3}$Δ/"

All strains are derivatives of SK1, except for BY4741, which is derived from S288C. SKY4016 is the strain whose genome was sequenced and assembled by the SGRP. Other strains are derived from the SK1 lineage in the Kleckner laboratory.

Plasmid pMS32 was used to delete $Ty_{CGR1\text{-}SCW11}$ and constructed as follows. PCR primers used to amplify regions of interest are shown in Table S3. $Ty_{CGR1\text{-}SCW11}$–L (~1.4-kb upstream of $Ty_{CGR1\text{-}SCW11}$) was digested with XbaI and BamHI and subcloned into corresponding sites of pRS306. Then, $Ty_{CGR1\text{-}SCW11}$–R (~0.8-kb downstream of $Ty_{CGR1\text{-}SCW11}$), digested with BamHI and HindIII, was subcloned in corresponding sites of pRS306 containing $Ty_{CGR1\text{-}SCW11}$–L. A $Ty_{CGR1\text{-}SCW11}$ was replaced with a BamHI site by two-step gene replacement, using pMS32 linearized with BamHI. Similarly, a $Ty_{EST3\text{-}FAA3}$ was replaced with a EcoRI site, using EcoRI-digested pMS35, which contains $Ty_{EST3\text{-}FAA3}$–L (~1.5-kb upstream of $Ty_{EST3\text{-}FAA3}$) and $Ty_{EST3\text{-}FAA3}$–L (~0.9-kb downstream of $Ty_{EST3\text{-}FAA3}$), which flank a EcoRI recognition sequence. Two independent diploid clones of *dmc1*Δ $Ty_{CGR1\text{-}SCW11}$Δ (SKY4188 and 4189) and of *dmc1*Δ $Ty_{EST3\text{-}FAA3}$Δ (SKY4191 and 4192) were used for physical DSB analysis (Figure 6).
