# Supplementary material for: Meiotic Recombination Initiation in and around Retrotransposable Elements in Saccharomyces cerevisiae
Source: PLoS Genet. 2013 Aug 29;9(8):e1003732. doi: 10.1371/journal.pgen.1003732 (PMC3757047; doi:10.1371/journal.pgen.1003732)
Supplement: Table S3 — Primers used in this study. (PDF) [file pgen.1003732.s004.pdf]

Table S3. Primers used in this study

Region	Sequence
Ty$_{CGR1\text{-}SCW11}$–L[a]	5′-GCTCTAGATGACTCTGATATCTTGACCACCTTGGC<br>5′-GCGGATCCATTATAACATTCAGGTAATTATCAATTTTTATTTTTTAGAATGG
Ty$_{CGR1\text{-}SCW11}$–R[a]	5′-CGGGATCCATACAAAGTGAAATGATAATGTAGATATGTAAATGAACTGAATAATCCCG<br>5′-CATAAGCTTTGGAAATGAAGCTATCAATAATGGTTGG
Ty$_{EST3\text{-}FAA3}$–L[a]	5′-CGAGGATCCCACCAGAAGTTAGTGTAAATGACGCC<br>5′-CCGGAATTCGAACATGAAAAGTAGTAGAAAAATCAGC
Ty$_{EST3\text{-}FAA3}$–R[a]	5′-GACGAATTCGTTTTTCAATAATTTGCAAGTTATATATCTTTGCC<br>5′-AGTCCAAGCTTCGGGACCCCTCCGAAGGG
Ty$_{URA3}$[b]	5′-CGAAGGAAGGAGCACAGAC<br>5′-GCTCTAATTTGTGAGTTTAG
Ty$_{YMR118C\text{-}ASI1}$[b]	5′-GATCGGACTGCGTTCAGTGAACC<br>5′-CGCCCTCAAAGGAATACGCG
PEX25 probe[c]	5′-ACGGCATACGTTCCCTTTGG<br>5′-GCACACCATTCCGAGTGTGC
DOT5 probe[c]	5′-TGAAATCCACAGACGGCTGTGC<br>5′-GGCGTCATTTACACTAACTTCTGGTG
ESP1 probe[c]	5′-GCGCCTGTACTGTTCCTCGTG<br>5′-GAGGGGTTATAAAGTCTAATTTCAGGG
RPS24A probe[c]	5′-CATGGTTGTTCAGGCGTTCGC<br>5′-TGAGCTTTGCCATTCCGTCTCC
CWH41 probe[c]	5′-CAGTACCATGACCATCTATCGGACTG<br>5′-CTGGCTTGTTAACCGAATATGCC
GEA2 probe[c]	5′-GACAGAGGCGGCAATATTACTCGAC<br>5′-GCCTGAAGAGCACCACGGC
RCS6 probe[c]	5′-TGCAGAGGCAGAATCCAGGG<br>5′-TGGGAGTAGAGTAGTGAGATATTGCCC
YCR057C probe[d]	5′-ATGCTGTCACTACACGTGAACC<br>5′-CAGACAGTTTGCTCCCTTCG
YKL182W probe[d]	5′-TCAAGCCAAGCCAAGGAATGG<br>5′-GAAACAACATCACCAACTTGCAG

[a] Primers used to construct plasmids pMS32 and pMS35 (see the legend to Table S2).
[b] Primers used to confirm presence of Ty$_{URA3}$ and Ty$_{YMR118C\text{-}ASI1}$ (Figures 2C and 2D) are shown. Primers for other Ty insertions are available upon request.
[c] Primers used to prepare probes for Southern blotting.
[d] Primers used to prepare control probes in Figure 6 (see also Materials and Methods).
